# Skin Lesions on Common Bottlenose Dolphins (*Tursiops truncatus*) from Three Sites in the Northwest Atlantic, USA

**DOI:** 10.1371/journal.pone.0033081

**Published:** 2012-03-12

**Authors:** Leslie Burdett Hart, Dave S. Rotstein, Randall S. Wells, Jason Allen, Aaron Barleycorn, Brian C. Balmer, Suzanne M. Lane, Todd Speakman, Eric S. Zolman, Megan Stolen, Wayne McFee, Tracey Goldstein, Teri K. Rowles, Lori H. Schwacke

**Affiliations:** 1 Division of Biostatistics and Epidemiology, Department of Medicine, Medical University of South Carolina, Charleston, South Carolina, United States of America; 2 National Oceanic and Atmospheric Administration, National Ocean Service, Hollings Marine Laboratory, Charleston, South Carolina, United States of America; 3 National Oceanic and Atmospheric Administration, National Marine Fisheries Service, Office of Protected Resources, Silver Spring, Maryland, United States of America; 4 Marine Mammal Pathology Services, Olney, Maryland, United States of America; 5 Chicago Zoological Society, c/o: Mote Marine Laboratory, Sarasota, Florida, United States of America; 6 University of North Carolina Wilmington, Department of Biology and Marine Biology, Wilmington, North Carolina, United States of America; 7 Hubbs-SeaWorld Research Institute, Melbourne Beach, Florida, United States of America; 8 Coastal Marine Mammal Stranding Assessments Program, National Oceanic and Atmospheric Administration, National Centers for Coastal Ocean Science, Center for Coastal Environmental Health and Biomolecular Research, Charleston, South Carolina, United States of America; 9 Wildlife Health Center, School of Veterinary Medicine, University of California, Davis, California, United States of America; University of Iowa, United States of America

## Abstract

Skin disease occurs frequently in many cetacean species across the globe; methods to categorize lesions have relied on photo-identification (photo-id), stranding, and by-catch data. The current study used photo-id data from four sampling months during 2009 to estimate skin lesion prevalence and type occurring on bottlenose dolphins (*Tursiops truncatus*) from three sites along the southeast United States coast [Sarasota Bay, FL (SSB); near Brunswick and Sapelo Island, GA (BSG); and near Charleston, SC (CHS)]. The prevalence of lesions was highest among BSG dolphins (*P = 0.587*) and lowest in SSB (*P = 0.380*), and the overall prevalence was significantly different among all sites (*p<0.0167*). Logistic regression modeling revealed a significant reduction in the odds of lesion occurrence for increasing water temperatures (*OR = 0.92; 95%CI:0.906–0.938*) and a significantly increased odds of lesion occurrence for BSG dolphins (*OR = 1.39; 95%CI:1.203–1.614*). Approximately one-third of the lesioned dolphins from each site presented with multiple types, and population differences in lesion type occurrence were observed (*p<0.05*). Lesions on stranded dolphins were sampled to determine the etiology of different lesion types, which included three visually distinct samples positive for herpesvirus. Although generally considered non-fatal, skin disease may be indicative of animal health or exposure to anthropogenic or environmental threats, and photo-id data provide an efficient and cost-effective approach to document the occurrence of skin lesions in free-ranging populations.

## Introduction

Skin lesions in delphinids and other small cetaceans are geographically widespread [1]–[9]. Reported prevalence estimates for skin lesions among delphinids range between 48% [4] and 100% [3], and 63% to 100% for bottlenose dolphins (*Tursiops truncatus*) specifically [2], [3], [6], [8], [10], [11]. Tattoo lesions have been studied extensively by Van Bressem et al. [5], [9], [12], where the reported prevalence of this specific lesion type among bottlenose dolphins ranges from 0% [9] to 71% [12]. Most of these studies used photo-identification (photo-id) data to estimate the population prevalence of skin disease (e.g. [2], [3], [6], [8], [10]–[12]), producing minimum disease prevalence estimates [11] as the detection of lesions are restricted to body parts that are routinely photographed. Although limited for determining skin disease causes, photo-id data provide a relatively inexpensive and non-invasive means of assessing body and skin condition [13], as well as lesion progression, recurrence, or resolution [9], [14], [15] for free-ranging marine mammal populations. Other studies have relied on by-catch or stranding data [1], [4], [5], [9], [16], [17], or capture-release health assessment data [3], [18], [19] to estimate disease burden in wild populations; however, these methods can be limited by small sample sizes or in the case of stranding data, biased towards individuals with compromised health [20].

Microscopic evaluations of skin lesions among cetaceans have revealed a broad spectrum of causes including potentially infectious pathogens such as poxvirus [21] and herpesvirus [22], and lacaziosis (i.e. lobomycosis, [23]), as well as non-infectious causes such as diatom attachment [24] and traumatic scarring [1]. As skin lesions in wild dolphin populations may indicate the emergence [25] or persistence of infectious disease, detection and monitoring of lesions using photo-id data facilitate disease surveillance. Some studies have suggested an association between the presence of skin disease and environmental parameters (eg. salinity and temperature [3], [21]) or anthropogenic factors (eg. contaminants and pollutants [5], [6], [26]); therefore, assessing differences between populations could possibly indicate changes in environmental conditions or exposure to chemical contaminants.

**Figure 1:**
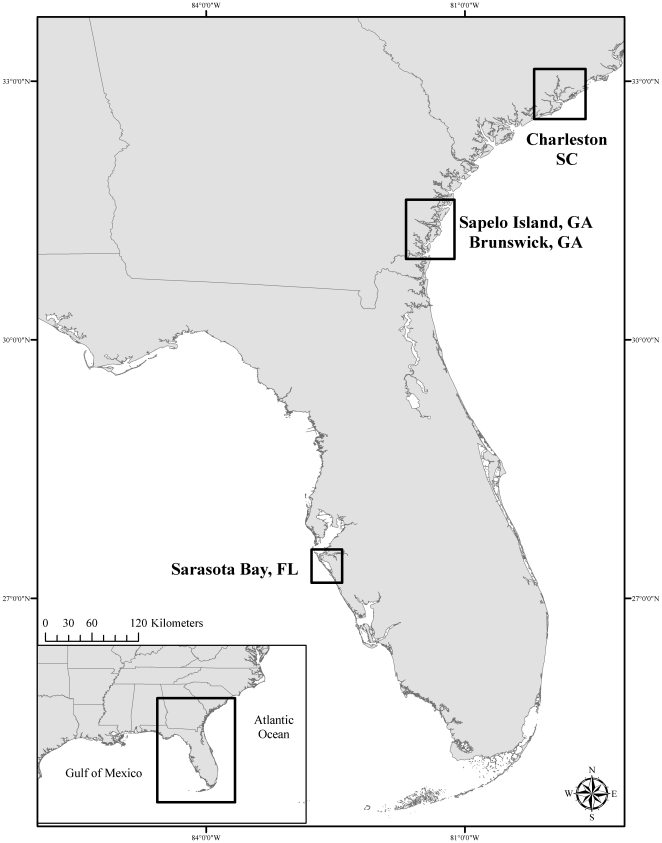
Location of photo-id study sites: Charleston, SC (CHS), Brunswick and Sapelo Island, GA (BSG), and Sarasota Bay, FL (SSB).

The objective of this study was to use photo-id data to estimate and compare the prevalence of skin lesions and lesion type among dolphins from three U.S. southeast coastal sites: Sarasota Bay and vicinity, Florida (SSB); near Brunswick and Sapelo Island, Georgia (BSG); and near Charleston, South Carolina (CHS)(Figure 1). These efforts provided an assessment of skin lesion occurrence and the distribution of different lesion types among dolphins from different geographic sites, which can serve as a baseline for future surveillance of novel types or changes in overall skin lesion prevalence.

## Materials and Methods

### Ethics Statement

Samples from stranded animals were collected under NOAA's responsibility to the MMPA 1972 under Section 109(h), and a Stranding Agreement as part of the Marine Mammal Health and Stranding Response Act to respond to and collect samples from stranded marine mammals. Photo-id surveys were conducted under the following NMFS Permit Numbers:

Sarasota Bay: 522–1785; Charleston, SC and Brunswick/Sapelo Island, GA: GA LoC No. 1064-1748 and GA LoC No. 14348.

### Study Populations

#### Sarasota Bay, FL (SSB)

The bottlenose dolphin community in Sarasota Bay, FL (Figure 1) is comprised of approximately 160 residents [27], and has been monitored and studied since 1970. Routine photo-id surveys were initiated in 1980 [28] to estimate abundance [29], identify individuals and group composition [30], monitor social structure and life history [31], [32], and determine movement patterns [33].

#### Brunswick and Sapelo Island, GA (BSG)

Bottlenose dolphins in estuarine waters near Brunswick and Sapelo Island, GA (Figure 1) have been studied since 2004 [34]. A systematic photo-id study of dolphins in coastal GA waters was conducted during four seasons from 2008 to 2009, resulting in preliminary abundance estimates of less than 100 individuals during fall months to over 230 individuals during the summer in the Brunswick area, and approximately 150–350 individuals in waters near Sapelo Island depending on time of year [35].

#### Charleston, SC (CHS)

Photo-id studies of bottlenose dolphins inhabiting the Charleston, SC (Figure 1) estuarine system have been conducted on a semi-routine basis since 1994 [36]; monthly surveys were initiated in January 2004 [37]. Abundance estimates calculated from post-2004 surveys ranged from approximately 300 dolphins in January to 900 individuals in July due to an influx of animals into the survey area during the spring and summer months [37].

### Skin Lesion Detection and Classification

Digital photo-id images for the three sites were obtained across four seasons corresponding to the months of February, April, July, and October, 2009. All images from each dolphin sighting were visually screened for dolphins with skin lesions, based on the detection of a lesion on any dorsal or dorsolateral aspect of the animal's body. Cetacean photo-id projects utilized a standardized and quantifiable methodology [38] to select for the best images of an individual animal's dorsal fin (e.g. [37]). This methodology was modified for this study to detect skin lesions on individual dolphins. Digital images were excluded from lesion screening if distinguishing features of the lesions could not be observed (i.e. dark or backlit, in poor focus). The photographic quality of each sighting was scored as: 1) good – high confidence in determining the presence or absence of skin lesions for all or most animals in the sighting; 2) average – reduced confidence in determining the presence or absence of skin lesions for several animals in the sighting; 3) poor – no confidence in determining the presence or absence of skin lesions for any animal in the sighting. All images scored as ‘poor’ were excluded from final analyses, as well as images with lesions whose distinguishing features could not be detected.

Once all photos were screened for lesions, each lesion was categorized according to descriptions in previous studies [5], [9], [11], [12]: 1) black; 2) pale; 3) cloudy; 4) lunar; 5) dark-fringed; 6) white-fringed; 7) orange patches; 8) tattoo-like; 9) white velvety; 10) lacaziosis-like; or 11) vesicular. In addition to these lesion types, two new categories, ‘spotted’ and ‘mottled’, were added, resulting in 13 possible lesion categories. Spotted lesions were defined as having localized or widespread distribution, were paler in color than the surrounding skin, circular in shape, and did not have a dark border. Mottled lesions were defined as scattered flecks of white, pale gray, or dark gray pigmentation, irregularly shaped, and were usually located laterally.

### Dolphin Identification

All dolphins screened for skin lesions were identified as unique individuals and matched to known animals in a photo-id catalog based upon distinctive markings located on the dorsal fin. The methodology for dorsal fin identification has been described elsewhere [39]. Individuals with marginally distinct and non-distinct dorsal fins were excluded from the analysis to avoid inadvertent duplication of records.

### Stranding Sampling

**Figure 2:**
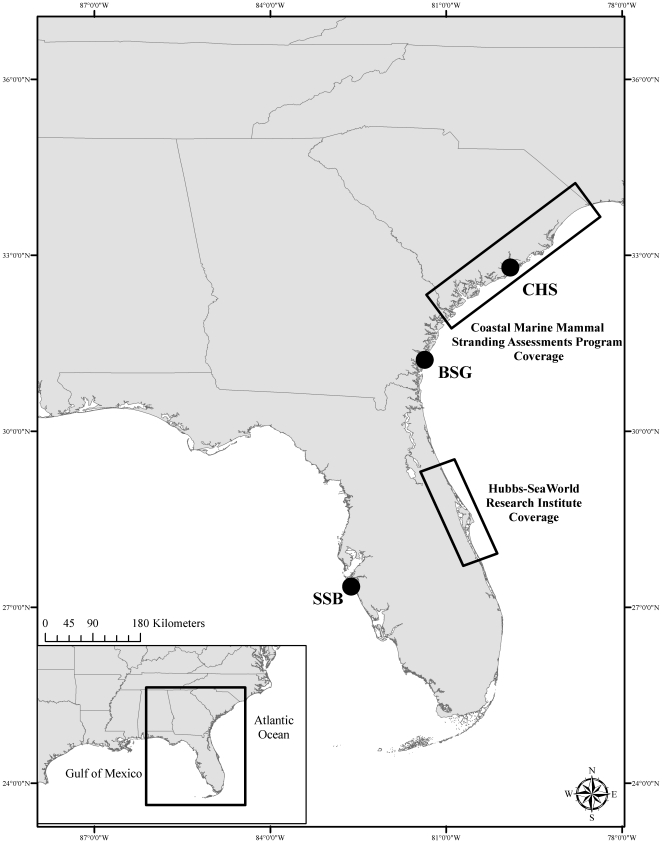
Photo-id study sites [Charleston, SC (CHS); Brunswick and Sapelo Island, GA (BSG); Sarasota Bay, FL (SSB)] and stranding coverage areas for the Coastal Marine Mammal Stranding Assessments Program (South Carolina) and Hubbs-SeaWorld Research Institute (Florida).

To supplement the findings from the photo-id assessment of lesion occurrence and to help examine etiology, lesion samples, categorized according to the aforementioned descriptions, were opportunistically obtained from two stranding organizations in the southeast United States (Coastal Marine Mammal Stranding Assessments Program, Charleston, SC and Hubbs-Sea World Research Institute's Marine Mammal Strandings Program, Melbourne Beach, FL) between 2008 and 2010. These organizations were selected as their stranding response covers geographic areas that are in close proximity to the photo-id study sites (Figure 2); however, overlap of study subjects between the photo-id and stranding assessments was not an intended objective. Lesion sites were swabbed for bacterial culture, and individual lesion biopsies were sub-sampled and preserved in: 1) 10% buffered formalin; 2) viral transport media; 3) RNA Later® (Applied Biosystems/Ambion, Austin, TX); and 4) frozen whole at −20°C. Histological analyses were conducted by a veterinary pathologist, and samples revealing evidence of a viral source were submitted for polymerase chain reaction (PCR) analyses (e.g. [40]–[42]) to the Wildlife Health Center at the University of California (UC Davis) School of Veterinary Medicine.

DNA was extracted from the skin tissues using a commercial kit according to manufacturer's instructions (DNeasy Blood and Tissue Kit, Qiagen Inc.,Valencia, CA). Multiple primers were used to test for the presence of poxviral DNA. PCR was performed to detect a 594 bp fragment of the genomic region encoding for the virion envelope antigen (p42K) of parapox viruses [42] and a 344 bp fragment of the hemagglutinin (HA) protein of orthopox viruses [40]. PCR to detect herpesviral DNA was performed using primary and nested consensus primers for the DNA-dependent-DNA polymerase (Dpol) gene of herpesviruses to amplify a fragment of 250 bp [41]. Parallel reactions amplifying a 350 bp fragment of the mammalian ferritin gene were performed to control for the PCR amplificability of the DNA sample. PCR products were resolved on 1–1.5% agarose gels. Bands of expected size were excised, purified using the QIAquick Gel Extraction Kit (Qiagen Inc.) and cloned (pCR4-TOPO vector; Invitrogen) and sequenced using the 3730 DNA Analyzer (Applied Biosystems).

Raw sequences were edited with Geneious Pro (version 5.1.4; Biomatters) [43], and the identity of the herpesviral DNA fragments were confirmed with the use of the BLASTn program run on the non-redundant National Center for Biotechnology Information (NCBI) database (http://blast. ncbi. nlm.nih.gov/Blast.cgi). Primer sequences were edited out prior to further analyses and phylogenetic analysis was performed to compare the obtained herpesviral sequences with 23 other herpesviral sequences from all three subfamilies. The nucleotide sequences of the DNA polymerase gene fragments were aligned using MUSCLE [44]. Bayesian analysis of the alignment was performed using Mr. Bayes 3.1 with gamma distributed rate variation [45]. Four incrementally heated Markov Chains were run for 1,100,000 generations, sampling every 200 generations, where 10% of 1,100,000 iterations were discarded as burn in. IgHv-2 (Iguanid herpesvirus-2, Genbank accession number AY236869) was used as the outgroup due to its early divergence from other herpesviruses.

### Statistical Analysis

The prevalence of skin lesions was estimated by the proportion of distinct individuals with at least one skin lesion compared to all distinct individuals sighted during the study period (i.e. 2009 overall or each month). Individuals with and without skin lesions during two or more months of the study were only counted once for the 2009 estimate. Similarly, if an individual was sighted with skin lesions twice during the same month, only one occurrence of the lesion was used to estimate the monthly prevalence of skin disease. The overall prevalence of skin lesions was compared between sites using a Chi Square test and Bonferroni correction for multiple comparisons (*α = 0.0167*; [46]), assuming the requirements for a normal distribution approximation [46], [47].

Using logistic regression analyses, the occurrence of skin lesions among dolphins from all three sites was examined relative to salinity (ppt) and water temperature (°C) measurements that were collected at each sighting used for lesion screening. Each individual was coded with a “1” (skin lesion present) or “0” (no skin lesion present). If an individual dolphin was sighted on more than one day in the month, salinity and temperature measurements were averaged for the multiple sightings. PROC LOGISTIC in SAS® (SAS Institute Inc., Cary, NC), was used to examine the associations *(α = 0.05)* between lesion occurrence and salinity and temperature where skin lesion status (0,1) was the dependent variable, temperature and salinity were continuous independent variables, and site (CHS, BSG, SSB) was a categorical variable.

Prevalence of different skin lesion types was estimated as the proportion of individuals with a particular lesion type relative to the total number of individuals with lesions, for a given time period (i.e. 2009 overall or by month). In some cases, animals presented with multiple lesion types. Within a single study site, the prevalence of different skin lesion types were compared between months using a Chi Square or Fisher's Exact test depending on expected cell counts [46], and *post-hoc* analyses to determine differences in lesion type occurrence were evaluated using a SAS® macro for multiple comparisons that relied upon arcsine transformation of binomial data (*α<0.05*; [47]). Major lesion types were also compared between sites using a Chi Square test and the multiple comparisons macro (*α<0.05*; [47]).

## Results

### Overall Skin Lesion Prevalence

Digital images of 266 distinct individuals were suitable for lesion screening during the study period in Sarasota Bay (SSB). Of these, 101 animals (*P = 0.380; 95% CI: 0.321–0.441*) had visible skin lesions. In Brunswick and Sapelo Island, GA (BSG), images of 322 distinct individuals were suitable for lesion screening, and 189 (*P = 0.587; 95% CI: 0.531–0.641*) animals were found to have visible skin lesions. Photo-id images were suitable for 351 individuals from Charleston, SC (CHS), and 171 (*P = 0.487; 95% CI: 0.434–0.541*) of these presented with at least one skin lesion (Table 1). Pairwise Chi Square tests with a Bonferroni correction for multiple comparisons (*α = 0.0167*) revealed a significant difference in skin lesion prevalence between all three sites (SSB<BSG *p<0.0001*; BSG>CHS *p = 0.0107*; CHS>SSB *p = 0.0088*; Table 1). Monthly differences were significant for the comparisons of SSB to the other two sites (Table 1). A consistent seasonal trend in skin lesion occurrence was observed for all three sites with monthly prevalence estimates ranked from highest to lowest: 1) April; 2) February; 3) October; 4) July (Table 1). It's unlikely that this trend was dependent solely on water temperature, as more lesions were observed in April than February. For CHS and BSG dolphins, lower lesion prevalence in the months of July and October could be related to an influx of animals during summer and fall [35], [37], assuming that non-residents were largely lesion-free. To better understand the potential effect of influx, dolphin composition in CHS was examined for the month of July. Of the 163 dolphins screened for lesions (Table 1), 47% were considered non-residents. Twenty-five percent of non-residents were observed with at least one skin lesion, while 18% of residents were lesioned, and these proportions were not significantly different based on a Chi-Square test of binomial proportions (*p = 0.3345*). Since one quarter of the non-residents were observed with lesions, and because the proportion of lesioned non-residents was greater than residents, it seems unlikely that the influx of non-resident dolphins to CHS in July was responsible for deflating lesion prevalence.

**Table 1:** Skin lesion prevalence and 95% CI for bottlenose dolphins photographed in waters near Charleston, SC (CHS), near Brunswick and Sapelo Island, GA (BSG), and Sarasota Bay, FL (SSB) in 2009.

	2009 Overall	February	April	July	October
**Sarasota Bay (SSB)**					
Photographed	266	151	127	124	132
AWL	101	40	45	18	20
Prevalence	0.380	0.265	0.354	0.145	0.152
95% CI	(0.321–0.441)	(0.197–0.343)	(0.272–0.444)	(0.088–0.220)	(0.095–0.224)
pvalue (SSB vs BSG)	<0.0001*	<0.0001*	<0.0001*	0.0066*	0.0041*
**Georgia (BSG)**					
Photographed	322	143	139	195	98
AWL	189	85	96	50	29
Prevalence	0.587	0.594	0.691	0.256	0.296
95% CI	(0.531–0.641)	(0.509–0.676)	(0.607–0.766)	(0.197–0.324)	(0.208–0.397)
pvalue (BSG vs CHS)	0.0107*	0.1296	0.1095	0.0653	0.1009
**Charleston (CHS)**					
Photographed	351	113	108	163	123
AWL	171	56	74	35	32
Prevalence	0.487	0.496	0.685	0.215	0.260
95% CI	(0.434–0.541)	(0.400–0.591)	(0.589–0.771)	(0.154–0.286)	(0.185–0.347)
pvalue (CHS vs SSB)	0.0088*	<0.0001*	<0.0001*	0.0400	0.0124*

* *Indicates a significant difference with a Bonferroni correction for multiple comparisons (α = 0.0167).*

*“Photographed” - number of dolphins evaluated for lesions with photographs suitable for lesion detection.*

*“AWL” - number of dolphins photographed with visible skin lesion(s).*

*“Prevalence” - proportion of ‘Photographed’ that is ‘AWL’.*

*(p values are reported for comparisons of skin lesion prevalence between sites).*

**Figure 3:**
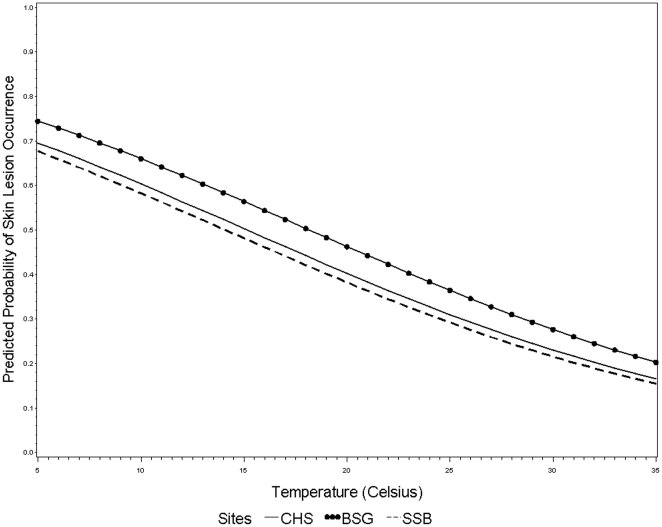
Regression curve for logistic model of the predicted probability of bottlenose dolphin (*Tursiops truncatus*) skin lesion occurrence using water temperature and study site as independent variables [Charleston, SC (CHS); Brunswick and Sapelo Island, GA (BSG); Sarasota Bay, FL (SSB)].

The minimum, maximum, and mean values for the salinity and temperature measurements were calculated for all three study sites. Mean water temperatures for CHS, BSG, and SSB were 21.1°C, 20.7°C, and 24.4°C, respectively. Mean salinities were 29.9 ppt (CHS), 25.1 ppt (BSG), and 33.1 ppt (SSB). Overall, the largest range in salinity (0.1–33.0 ppt) and water temperature (7.6–30.3°C) occurred in BSG. For all study sites, water temperature was associated with lesion occurrence (*p<0.05*); however, salinity was significantly associated with lesion occurrence only for the BSG animals. Therefore, the overall environmental logistic regression model for all three sites used water temperature and study site as dependent variables (Figure 3). This model indicated an 8% reduction in the odds of lesion occurrence for each unit increase (°C) in water temperature after adjusting for study site (*OR = 0.92; 95%CI: 0.906–0.938*), as well as significantly increased odds of skin lesions among dolphins in BSG (39%) after adjusting for water temperature (*OR = 1.39; 95%CI: 1.203–1.614*). It is possible that the site-specific difference in association between lesion occurrence and salinity was a result of the wide salinity range for BSG. Therefore, *post hoc* logistic regression modeling of lesion occurrence in BSG was conducted using both water temperature and salinity, revealing significant associations with both environmental variables (temperature: *OR = 0.92, 95%CI: 0.895–0.944;* salinity: *OR = 0.96, 95%CI: 0.938–0.922*).

### Skin Lesion Type

**Figure 4:**
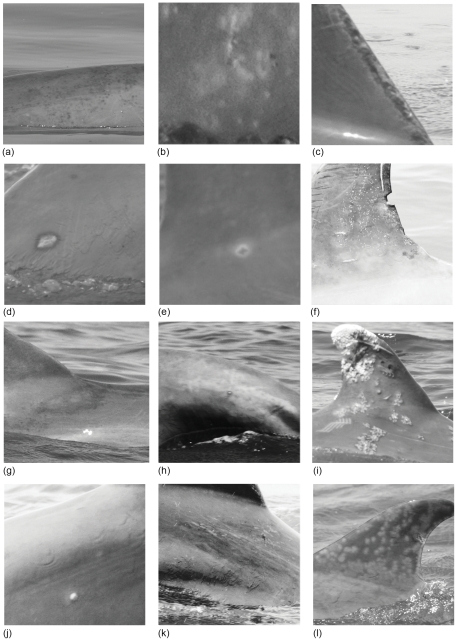
Examples of skin lesion types on free-ranging bottlenose dolphins (*Tursiops truncatus*) photographed in waters near Charleston, SC (CHS), Brunswick and Sapelo Island, GA (BSG), and Sarasota Bay, FL (SSB) in 2009. Lesion types include: black (a); pale (b); lunar (c); dark-fringed spots (d); white-fringed spots (e); orange patch (f); tattoo-like (g); white velvety (h); lacaziosis-like (i); vesicular (j); mottled (k); and spotted (l). (Categories from [3], [5], [9], [12]) Photo credit: B.Balmer, NCCOS/NOS/NOAA, Sarasota Dolphin Research Program.

Lesions representing 12 of the 13 possible categories were observed on dolphins in CHS, BSG, and SSB (Figure 4). Overall, Sarasota Bay dolphins presented with the greatest number of different skin lesion types (*n = 11*), while the fewest types occurred in Charleston (*n = 9*; Table 2). Approximately one-third of the dolphins from all three sites presented with multiple lesion types (*CHS = 0.38; BSG = 0.36; SSB = 0.30*). Cloudy lesions were not observed on dolphins from any of the photo-id study sites. Spotted lesions were only observed on BSG dolphins during July, and lacaziosis lesions were only present on SSB dolphins. The most common lesion type observed on dolphins from CHS and BSG were dark-fringed, whereas SSB dolphins were most commonly observed with tattoo-like lesions (Table 2).

**Table 2:** Prevalence of skin lesion types among lesioned dolphins photographed in waters near Charleston, SC (CHS), near Brunswick and Sapelo Island, GA (BSG), and Sarasota Bay, FL (SSB) in 2009.

Type	Charleston (n = 171)	Georgia (n = 189)	Sarasota Bay (n = 101)
**Black**	0.263	0.201	0.287
**Dark-Fringed**	0.550	0.577	0.238
**Lunar**	0.064	0	0.020
**Pale**	0.123	0.212	0.158
**Tattoo**	0.199	0.212	0.426
**Vesicular**	0.152	0.302	0.109
**White Velvety**	0.006	0.069	0.040
**White-Fringed**	0.146	0.116	0.030
**Lacaziosis-Like**	0	0	0.040
**Orange Patch**	0	0.069	0.069
**Mottled**	0.047	0.005	0.129
**Spotted**	0	0.053	0
**Cloudy**	0	0	0
**Other**	0.018	0.021	0.010

**Figure 5:**
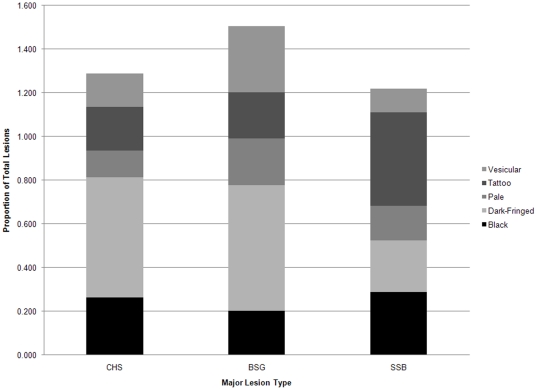
Proportion of major skin lesion types (2009 overall proportion >0.15) for free-ranging bottlenose dolphins (*Tursiops truncatus*) photographed in waters near Charleston, SC (CHS), near Brunswick and Sapelo Island, GA (BSG), and Sarasota Bay, FL (SSB) 2009.

Lesion types for which the prevalence among animals from a given site was greater than 15% were considered ‘major lesion types’ (Table 3, Figure 5). No significant differences in prevalence were observed between sites for black and pale lesions. The proportion of BSG and CHS animals with dark-fringed lesions was significantly higher than dolphins in SSB. Also, dolphins in BSG had a significantly higher prevalence of vesicular lesions than both CHS and SSB. The prevalence of tattoo-like lesions among SSB dolphins was significantly higher than among both BSG and CHS dolphins (*α<0.05*, Table 3).

**Table 3:** Proportion of major skin lesion types (*P>0.15*) and study site comparisons for lesioned dolphins photographed in waters near Charleston, SC (CHS), near Brunswick and Sapelo Island, GA (BSG), and Sarasota Bay, FL (SSB) in 2009.

Type	CHS (n = 171)	BSG (n = 189)	SSB (n = 101)	Chi Square (p)	Significant Difference∧
**Black**	0.263	0.201	0.287	0.1964	-
**Dark-Fringed**	0.550	0.577	0.238	<0.0001*	CHS>SSB BSG>SSB
**Pale**	0.123	0.212	0.158	0.0757	-
**Tattoo**	0.199	0.212	0.426	<0.0001*	SSB>BSG SSB>CHS
**Vesicular**	0.152	0.302	0.109	0.0001*	BSG>SSB BSG>CHS

* *Indicates a significant difference (α = 0.05).*

∧ *Indicates a significant difference after post-hoc multiple comparisons tests (α<0.05; Horne and Plaehn 2007).*

Due to the observed geographic disparities in dark-fringed, tattoo-like, and vesicular lesions, associations between these lesion types and environmental parameters were examined using previously described logistic regression methods. Water temperature, after adjusting for study site, was significantly associated (*p<0.05*) with the occurrence of dark-fringed, tattoo-like, and vesicular lesion types; whereas, salinity was only significantly associated with dark-fringed lesions and therefore not included in subsequent regression models. The regression models for dark-fringed, tattoo-like, and vesicular lesions revealed a respective 12%, 5%, and 16% reduction in the odds of occurrence for each unit increase (°C) in water temperature (*OR_dark-fringed_ = 0.88, 95% CI: 0.863–0.905; OR_tattoo-like_ = 0.95, 95% CI: 0.921–0.986; OR_vesicular_ = 0.84, 95% CI:0.809–0.877*). Also, compared to SSB animals and after adjusting for water temperature, dolphins in CHS and BSG had a significantly increased odds of dark-fringed lesions (*OR_CHS_ = 3.59, 95% CI: 2.227–5.784; OR_BSG_ = 3.47, 95% CI:2.165–5.576*), a significantly decreased odds of tattoo-like lesions (*OR_CHS_ = 0.07, 95% CI: 0.026–0.207; OR_BSG_ = 0.73, 95% CI:0.459–1.161*), and BSG dolphins were significantly more likely to have vesicular lesions (*OR = 2.61, 95% CI: 1.306–5.215*).

### Stranding Sampling

Stranded bottlenose dolphins were retrieved from coastal and estuarine shorelines of South Carolina and the central east coast of Florida, which includes the estuarine waters of the Indian River Lagoon and the oceanic beaches of the Atlantic Ocean (Figure 2). Twenty-nine stranded dolphins presented with at least one skin lesion, and 10 of these animals had multiple lesion types. Based on morphological analyses, none of the stranded animals from Florida or South Carolina were suspected to belong to the offshore ecotype [48]. Forty lesion samples were initially examined by histology, and 11 of the lesions were subsequently analyzed by PCR due to the suspicion of a viral etiology.

Lacaziosis-like disease occurred twice on dolphins from Florida; however, histological examination of biopsies from both animals confirmed infection by *L. loboi* in only one sample. Although histological analyses of lesions categorized as spotted, dark-fringed, and tattoo-like revealed evidence suggestive of a viral infection, all samples were negative for poxvirus and herpesvirus using PCR. Pale lesion histology revealed indications of: 1) healing process due to prior trauma; 2) ectoparasite attachment site; 3) prior viral infection; and 4) inflammation. Three lesion samples tested positive by PCR and sequence analysis of the obtained fragments with the degenerate primers confirmed the presence of herpesviral DNA. Phylogenetic analysis indicated that two sequences (from lesions categorized as pale and cloudy [11]) were identical to each other and to Delphinid Herpesvirus 1 (Genbank accession number AY952779), a gammaherpesvirus previously detected in bottlenose dolphins; and the third (from a white-fringed lesion sample) was identical to Delphinid Herpesvirus 3 (Genbank accession number AY757301), an alphaherpesvirus previously detected in bottlenose dolphins.

## Discussion

### Environmental and Anthropogenic Influences

These results indicate that geographic differences exist in the prevalence of skin lesions, as well as in the distribution and occurrence of different skin lesion types among dolphins from the three study sites. These differences could potentially be explained by variations in environmental parameters (i.e. temperature, salinity) or disparities in susceptibility due to anthropogenic contaminant exposure. For example, the Altamaha River, the third largest freshwater input into the Atlantic Ocean from North America [49], bisects the BSG field site. Thus, dolphins sighted predominantly in the waters surrounding the Altamaha River may be more susceptible to skin lesion types associated with freshwater input or runoff. Furthermore, a previous study [3] reported an association between lesion occurrence and environmental factors, where the prevalence of skin lesions decreased with increasing water temperature and salinity. Results from the present study further support the link between the presence of skin lesions and colder water temperatures for all three study sites examined, as logistic regression analyses indicated a decreased odds of lesion occurrence with increasing water temperature. The highest prevalence of skin lesions for all three sites occurred in April, and the mean water temperature for all three sites in April was colder than for the months of July and October; however, February water temperatures were colder than April and the prevalence of skin lesions was lower. These results suggest that skin lesion occurrence is influenced by factors other than just water temperature, that there is a lag time between exposure to colder water temperatures and the clinical manifestation of disease, or that pathogen viability and dolphin susceptibility may be heightened when water temperatures are within a particular range. In this particular study, colder water temperatures in the northern study sites (CHS and BSG), and freshwater exposure to BSG dolphins could help to explain observed geographic differences in skin lesion prevalence.

As for differences in susceptibility resulting from chemical contaminant exposure, recent analyses of remote and surgical blubber biopsies of dolphins from Brunswick, GA revealed unprecedented levels of polychlorinated biphenyls (PCBs) [34], [50]. Previous studies of PCB toxicity among mammalian laboratory animals indicated that skin lesions can manifest from exposure to different contaminant mixtures [51]–[53]. Anthropogenic contaminants have also been linked to immune suppression in marine mammals [54]–[56]; therefore, high levels of PCB exposure among BSG dolphins could contribute to the higher prevalence of skin lesions among animals from this site due to an inability to ward off pathogens.

### Lesion Identification and Differentiation

Three visually distinctive lesions from stranded dolphins were found positive for herpesvirus by PCR. Furthermore, two of the samples were found positive for identical strains, which suggest that different lesion types are not always representative of different diseases. Histological evidence of poxviral infection has been observed in biopsies of tattoo lesions [17], [21], as well as in ‘ring lesions’ [21], [57] and ‘round marks’ [4], which included dark-fringed spots as described in the current study. Although all dark-fringed and tattoo-like samples in this study were negative for poxviral infection by PCR, histological analyses provided evidence of a prior viral infection (i.e. cytoplasmic swelling, intracytoplasmic inclusion bodies). Results from the current study cannot confirm if poxvirus is associated with dark-fringed or tattoo-like lesions from the sampled dolphins; however, it seems possible that the virus could manifest as both lesion types. These stranding results strengthen the hypothesis that different visual lesion types could be caused by the same pathogen or may be different stages of the same disease [3], [4], [11].

### Bottlenose Dolphin Skin Lesion Differences between Study Sites

The prevalence of skin lesions among animals from the three study sites ranged from 38% to 59%. These estimates are substantially lower than the prevalence of skin lesions reported in other bottlenose dolphin populations across the globe, which ranged between 63% and 100% [2], [3], [6], [8], [10], [11]. Skin lesion occurrence has not been previously assessed for the CHS or BSG dolphins, so it is possible that the prevalence values reported here are minimum estimates for animals from these study sites. However, in a previous study, 62.7% of Sarasota Bay dolphins had at least one skin lesion detected on the dorsal fin [3]. The discrepancy between the previous and the current SSB prevalence estimate (38.0%) could be explained by a decreased burden of skin disease among dolphins from the SSB site during the intervening decade, differences in lesion categorization, or differences in photographic quality between studies. The lesion categories for both studies were derived from previous descriptions [11]; however, the current study did not include orange hues as a skin lesion type as the focus was on lesions that may have an infectious disease etiology, and orange hues could be films caused by the external attachment of diatoms [24]. In the Wilson et al. study [3], orange lesions were included in the ‘other’ category, which comprised 42% of the lesions observed in SSB; therefore, it is possible that the exclusion of animals with orange hues may have contributed to the lower prevalence estimate for skin lesions in SSB. As for differences in image quality between the two studies, higher-resolution digital images that were used for the current study likely improved the discretion of epidermal markings resulting from scarring or trauma that may have previously been classified as a skin lesion in lower resolution photographs. Although the prevalence of skin lesions on SSB dolphins was substantially different than a previous study [3], both estimates of skin lesion prevalence for SSB dolphins was the lowest compared to all other sites examined in either study.

In addition to environmental and anthropogenic influences, differences in skin lesion prevalence among dolphins from the three study sites in this paper could be due to heterogeneous age-class and sex distributions. Several previous studies of skin lesions and cetaceans have indicated differential susceptibility and severity among animals of varying age-class [9], [12], [17] and sex [8]. Age and sex data were not uniformly available for animals examined in the current study, and analyses that rely upon photo-id data are often limited to images of dorsal body surfaces, which prevent sex determination from genital morphology. Furthermore, age-class identification from photo-id data is often limited to adult or calf distinctions, which may not provide useful information for diseases that commonly occur among sub-adults (e.g. tattoo skin disease [9], [17]). To obtain a clear epidemiological understanding of factors influencing the prevalence of skin lesions and different lesion types, age-class and sex information are necessary.

### Conclusion

Skin lesions among bottlenose dolphins are geographically widespread and can affect a large proportion of a population. Also, lesion types may be differentially distributed among populations. In the current study, the prevalence of skin lesions was significantly different among dolphins from the three study sites, and differences in the occurrence of lesion types were also observed. The findings suggest that skin disease can vary by population, and that certain disease types may be geographically distinct. These geographic differences may be due to seasonal or environmental fluctuations, exposure to anthropogenic influences, or differences in population demographics; however, more research in these areas is needed to confirm this. This study demonstrates that images from photo-id surveys can be used as a non-invasive and cost-effective approach to study lesion occurrence in wild cetacean populations, and while many skin lesions do not appear to be fatal [2], [12], [21], [57], [58], lesions detected on free-ranging animals may serve as an indication of other underlying health concerns or environmental threats.
